# Reanalysis shows there is not an extreme decline effect in fish ocean acidification studies

**DOI:** 10.1371/journal.pbio.3001809

**Published:** 2022-11-22

**Authors:** Philip L. Munday

**Affiliations:** ARC Centre of Excellence for Coral Reef Studies, James Cook University, Townsville, Australia

## Abstract

This Formal Comment uses re-analysis after appropriate corrections to claim that the extreme decline effect reported by Clements et al. is a statistical artefact caused by the way they corrected for zeros in percentage data, exacerbated by errors in data compilation, selective data inclusions and missing studies with strong effects.

Clements and colleagues [1] claim there is an extreme decline effect in studies published between 2009 and 2019 on the impacts of ocean acidification (OA) on fish behaviour, with the modelled average effect size declining from >5 in 2009 to 2010 to <0.5 after 2015. Here, I show that the extreme decline effect reported by Clements and colleagues is a statistical artifact caused by the way they corrected for zero values in percentage data, which was more common in the earliest experiments compared with later studies. Furthermore, selective choices for excluding or including data, along with data compilation errors and missing studies with strong effects, weakened the effect sizes reported for papers after 2010, further exacerbating the decline effect reported by Clements and colleagues. When the data is reanalysed using appropriate corrections for zeros in percentage and proportional data and using a complete, corrected, and properly screened data set, the extreme decline effect reported by Clements and colleagues no longer exists (Fig 1A and 1B). Instead, there is a more gentle and consistent decline in effect size magnitude through time, from a modelled average <3 in 2009 to 2010 (Fig 1C) and remaining well above zero in 2018 to 2019 (Fig 1D).

**Fig 1:**
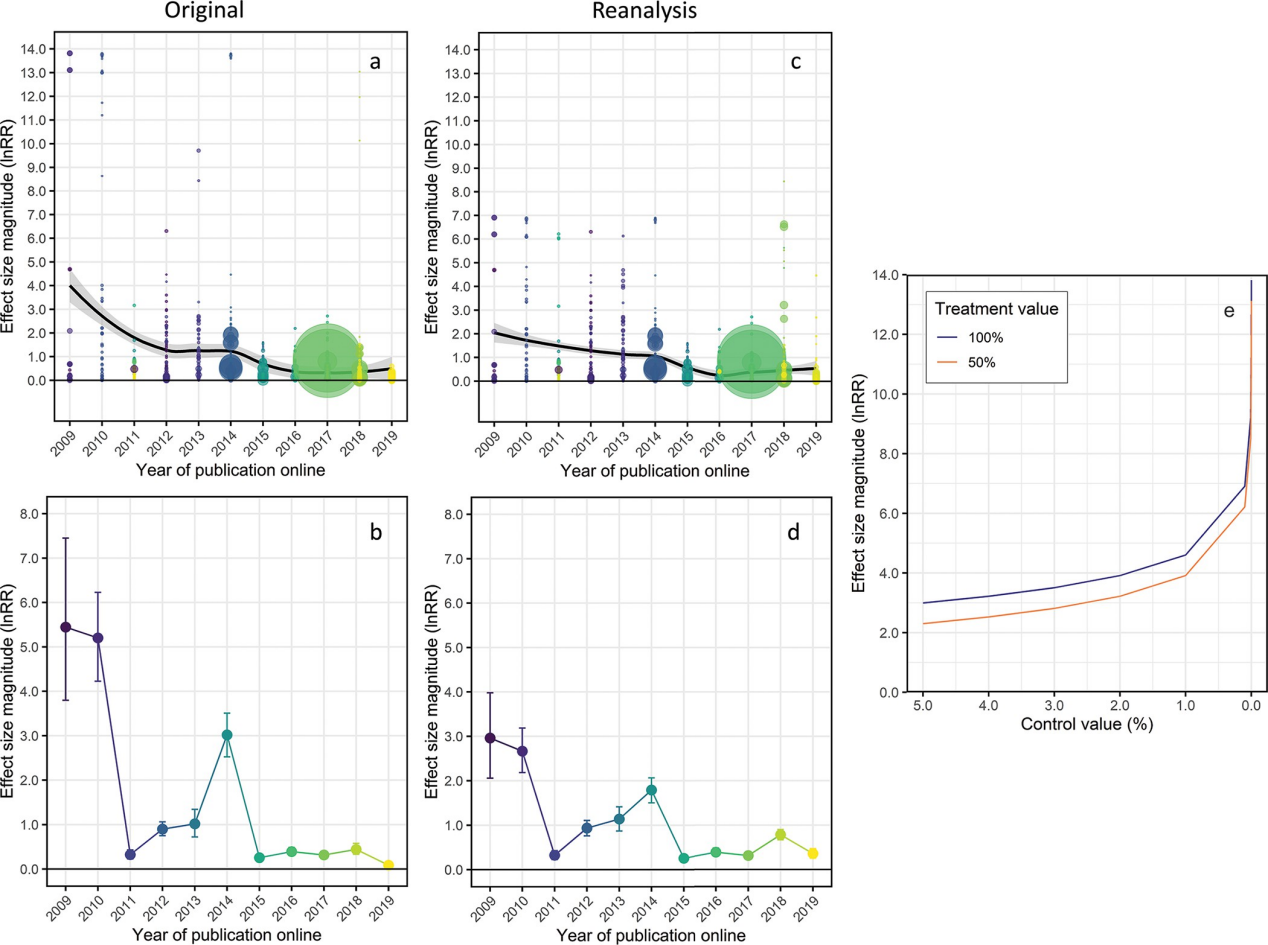
Reanalysis of effect sizes in studies on the impacts of ocean acidification on fish behaviour. (a, b) Original analysis by Clements and colleagues using 0.0001 to replace zero values in percentage and proportional data and (c, d) reanalysis with the corrected, updated, and screened data set using 0.1 to replace zero values in percentage data and 0.001 to replace zero values in proportional data. Top row (a, c) shows all calculated effect sizes (lnRR) fitted with a Loess curve and 95% confidence bounds. Bottom row (b, d) shows the modelled variance–weighted average effect sizes by year. Experiments with smaller variance are given greater weight in calculating the model means in the bottom row. Panel (e) shows how effect sizes are exaggerated when values below 1 are used to replace zeros in percentage data. The calculated effect size magnitude (lnRR = ln(treatment mean/control mean)) is shown for treatment means of 100% and 50% and control mean values between 5% and 0%, illustrating the inflation of lnRR caused by values below 1% in the denominator. The data underlying this figure (a–d) may be found in https://doi.org/10.25903/jw8m–9007. The data underlying panel (e) are found in S1 Data.

The primary reason for the extreme decline effect reported by Clements and colleagues is their decision to replace zero values in percentage data (range 0% to 100%) with a tiny value to 4 decimal places (i.e., 0.0001) to permit the calculation of a response ratio. Because lnRR is a ratio of the treatment mean/control, the use of an extremely small denominator results in an immensely inflated response ratio. The same applies if the numerator is extremely small; it produces a hugely inflated negative lnRR. The problem with using a small fractional value to replace zero values when calculating lnRR is illustrated in Fig 1E, which shows the steep increase in lnRR for increasingly small fractional values below 1. For example, if the control mean is 0% and the treatment is 100%, then: ln(99/1) = 4.6 if the smallest whole number (1) is used to replace zero values. lnRR increases 50% to 6.9 if 0.1 is used to replace zeros (ln(99.9/0.1) = 6.9) and doubles again to 13.8 using 0.0001 to correct for zeros (ln(99.9999/0.0001) = 13.8). In other words, lnRR becomes increasingly inflated as the size of the denominator decreases below 1 (Fig 1E). Clements and colleagues’ decision to replace zeros in percentage data with 0.0001 is especially perplexing when the resolution of the studies is considered. Measuring any fish behaviour to 0.0001% accuracy would be extraordinarily challenging. Moreover, of the 446 non-zero percentage values in the data set, only 5 are below 1, with the smallest being 0.56%, many orders of magnitude greater than the 0.0001% replacement value selected by Clements and colleagues.

Data simulations show how using 0.0001 to correct for zeros in percentage data exaggerates the decline effect. Using Clements and colleagues’ data set that has been corrected for data errors, screened for inappropriate inclusions (sham treatments and fluctuating CO_2_ treatments, see below) and with missing data included (Table A in S1 Text), Fig 2 shows how the decline effect is driven by the choice of replacement values used in percentage and proportional data. When zero values are replaced with 0.0001, the complete, corrected, and screened data set exhibits a decline in effect size that is not dissimilar to that originally reported by Clements and colleagues (Fig 2A and 2B), except that the initial decline is less steep (Fig 2C), and the variance-weighted average effect sizes are noticeably higher in 2018 to 2019 compared with the original (Fig 2D). However, the decline effect is markedly flatter (Fig 2E), and the magnitude of weighted average effect sizes in 2009, 2010, and 2014 are substantially smaller (Fig 2F) when 0.1 is used to correct for zero values in percentage and 0.001 for proportional data. The decline effect is even flatter (Fig 2G), and weighted effect sizes in 2009, 2010, and 2014 are smaller again (Fig 2H), when zero values in percentage data are replaced with the smallest whole number (1) and 0.01 for proportional data. From this comparison, it is clear to see that Clements and colleagues claim of an extreme decline effect is a statistical illusion driven by their method of correcting for zero values in percentage data. Indeed, Lajeunesse [2] warns that “log–ratio effect sizes estimated with RR are at the greatest risk of bias when: (1) the means have small sample sizes, (2) the two means are not close to one another, and (3) at least one of the control and treatment means is near zero” all of which apply here.

**Fig 2:**
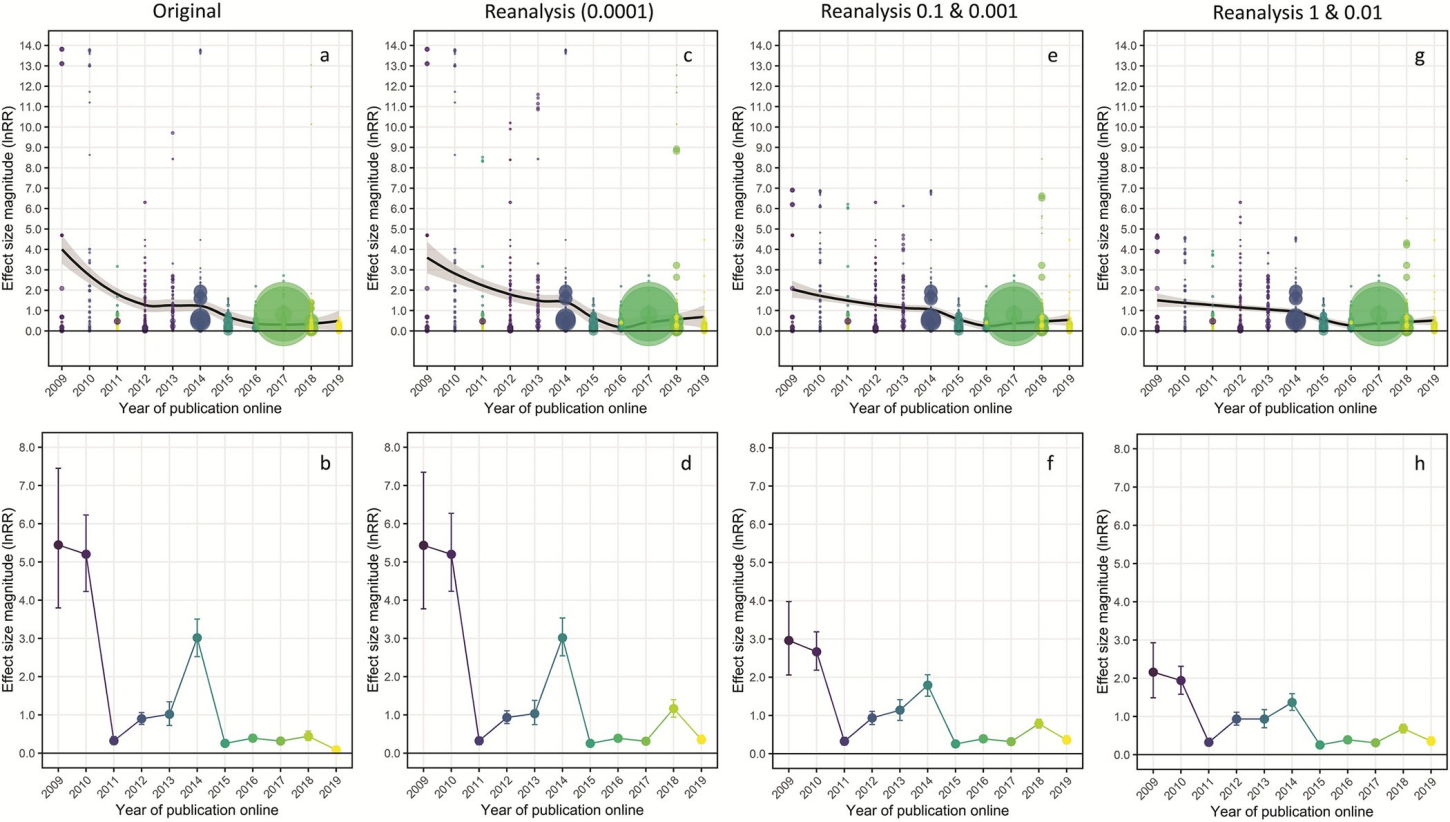
Comparison of decline effect and variance–weighted mean effect sizes with different methods of correcting for zero values in percentage and proportional data. (a, b) Original data from Clements and colleagues using 0.0001 and reanalysis with (c, d) corrected, updated, and screened data using 0.0001; (e, f) corrected, updated, and screened data using 0.1 for percentage data and 0.001 for proportional data; and (g, h) corrected, updated, and screened data using 1 for percentage data and 0.01 for proportional data. Data underlying this figure may be found at https://doi.org/10.25903/jw8m–9007.

Clements and colleagues [1] analysis also contains data handling errors, improper data inclusions and exclusions, and missing studies (Table A in S1 Text), all of which exaggerate the decline effect.

A preliminary check of Clements and colleagues’ data reveals data entry errors and incorrect values that cause effect sizes to be lower than the true value for studies after 2010. The feeding strikes data for McMahon and colleagues [3] does not match the figure or the underlying raw data, and there are errors in the reported *N* values, despite the correct data being publicly available online since publication. There are also mistakes in the coding of cue type and life stage of some studies (Table A in S1 Text), as well as incorrect values in the year of publication online and print columns for numerous files (see Methods in S1 Text). These mistakes illustrate how easy it is to make unintentional data handling errors in large, complex data sets, even by authors who have been highly critical of others for doing just that.

Another problem that artificially diminishes effects sizes in papers after 2010 is the inclusion of sham treatments in the calculation of OA treatment effect sizes. Sham treatments, such as the injection of blank seawater with no additional stimulus, are often used in studies that measure the change in behaviour after a stimulus (e.g., predator or alarm cue) is presented, compared to a prestimulus period. Sham treatments are predicted to have no or very small effects if an experiment is working properly. By including these methodological controls as experiments in their analyses, Clements and colleagues have artificially diluted the effect size for several studies conducted after 2010 (Table A in S1 Text). Yet, they removed procedural controls (tests with seawater on both sides of the flume) from studies in 2009 and 2010, thereby increasing the average effect size for those years.

Clements and colleagues also chose to exclude results where there was a different direction of responses between the control and the OA treatment (Table A in S1 Text). The problem here is that these are often the stronger results directly attributable to OA effects, precisely because the treatment effect goes in the opposite direction to the control. For example, the 3 species for which strong OA effects are observed at 850 ppm CO_2_ are excluded in the data set for Ferrari and colleagues [4], leaving only the 1 species that was found to be much more tolerant of elevated CO_2_ in the analysis. By excluding some of the strongest effects, while retaining weaker effects from the same experiments, Clements and colleagues have exacerbated the decline in effect size of experiments immediately after 2010.

A further issue is the inclusion of treatments that diminish the magnitude of OA effects, such as fluctuating CO_2_ treatments, which were not included in the original studies (Table A in S1 Text). For example, Jarrold and colleagues [5] showed that daily CO_2_ cycles greatly diminish the behavioural effects of OA compared with stable elevated CO_2_ treatments used in earlier studies. By including these treatments in their analysis, Clements and colleagues diminish the average effect size that would otherwise be attained.

Finally, some experiments and recent studies [6–7] with strong effects are missing from Clements and colleagues’ data set (Table A in S1 Text). Two studies [8–9] for 2019 are also missing, despite the figures portraying a full year. The absence of these studies causes the mean effect size estimated by Clements and colleagues for 2018 to 2019 to be lower than it should be (original versus reanalysis (0.0001) 2018: 0.443 versus 1.164, 2019: 0.088 versus 0.356). Moreover, the mean effect size in 2019 does not fall to zero when the data set is complete (Fig 1C and 1D).

Without doubt, there has been a decline through time in the averaged effect size from experiments investigating the behavioural effects of OA on fish, but it is not the extreme decline erroneously reported by Clements and colleagues. A decline in effect size is not surprising as more and different species are tested, some of which will be much less sensitive to the effects of OA than the orange clownfish, which was the first species tested in this field of study (e.g., [10]). Furthermore, an increasing range of different behaviours have been tested through time, many of which are less affected by OA and generate smaller effect sizes than the initial effects of OA on the response of larvae to highly concentrated predator odour and habitat cues. Methods have also changed through time, in ways that reduce effect sizes compared with the earliest studies in the field [11]. Decline effects occur in many areas of science, including ecology [12], yet their analysis and interpretation is still a work in progress [13–14].

## Supporting information

S1 Text Supporting information. (DOCX)

S1 Fig Effect sizes in studies on the impacts of ocean acidification on fish behaviour using only OA treatment levels ≥800 μatm CO_2_. (a) Calculated effect sizes (lnRR) fitted with a Loess curve and 95% confidence bounds and (b) modelled variance–weighted average effect sizes by year. The data underlying this figure may be found in https://doi.org/10.25903/jw8m–9007. Table A. Data errors identified in a non–exhaustive preliminary check of Clements and colleagues’ S2 data file, along with incorrect inclusion of sham treatments, missing data, and exclusions that were corrected to enable analysis. Highlight refers to the colour used to show the relevant lines of data in the screened, corrected, and complete data file used in the reanalysis. Data files available at https://doi.org/10.25903/jw8m–9007.
(DOCX)

S1 Data Data associated with Fig 1E. Calculated lnRR for control values ranging from 0.0001%–5% for treatment values of 50% and 100%.
(DOCX)
