# Supplementary material for: Reanalysis shows there is not an extreme decline effect in fish ocean acidification studies
Source: PLoS Biol. 2022 Nov 22;20(11):e3001809. doi: 10.1371/journal.pbio.3001809 (PMC9681065; doi:10.1371/journal.pbio.3001809)
Supplement: S1 Text — (DOCX) [file pbio.3001809.s001.docx]

Supporting Information: S1 Text

Methods

I manually screened Clements et al.’s S2 raw data file for errors, inappropriate inclusions and missing data (Table A). These are highlighted in red, blue and purple (respectively) in the data files available at Research Data JCU (https://doi.org/10.25903/jw8m-9007). The data errors I identified in Clements et al.'s data set were cross-checked and verified by an independent scientist.

Specific experiments excluded in Clements et al.’s S2 raw data file because there was a different direction of responses between the control and the OA treatment were adjusted to enable inclusion by replacing the small number in the OA treatment (or control) with a very small number of the same sign as the control (or OA treatment), as was done by Clements et al. for zero values throughout the data set. These corrections are highlighted in yellow in the data files available at https://doi.org/10.25903/jw8m-9007. This is a simple solution to the analytical problem of calculating lnRR when there is a different direction of response between the control and OA treatment.

**Table A**: Data errors identified in a non-exhaustive preliminary check of Clements et al.’s S2 data file, along with incorrect inclusion of sham treatments, missing data, and exclusions that were corrected to enable analysis. Highlight refers to the colour used to show the relevant lines of data in the screened, corrected and complete data file used in the reanalysis. Data files available at https://doi.org/10.25903/jw8m-9007.

| **Correction type** | **Highlight** | **Study** | **Rows in corrected data file** |
| --- | --- | --- | --- |
| Data errors | Red | a4 | 69, 70, 73, 74, 77, 78 |
|  | Red | a23 | 243, 244, 245, 246 |
|  | Red | a78 | 669, 670, 671, 672 |
| Incorrect inclusions | Blue | a11 | 120, 122, 124, 126, 128, 130, 132, 134, 136, 138, 140, 142, 144, 146, 148, 150, 153, 156 |
|  | Blue | a22 | 215, 218, 221, 224, 227, 230 |
|  | Blue | a27 | 285, 286, 289, 290 |
|  | Blue | a64 | 554, 545, 547, 548, 550, 552, 554, 556 |
|  | Blue | a65 | 558, 560, 562, 564, 566, 568 |
|  | Blue | a70 | 601, 602 |
|  | Blue | a78 | 665, 666, 667, 668 |
| Missing data | Purple | a23 | 247, 248, 249, 250, 251, 252 |
|  | Purple | a72 | 616, 617 |
|  | Purple | a92 | 828, 829 |
|  | Purple | a93 | 830, 831, 832, 833, 834, 835 |
|  | Purple | a94 | 836, 837, 838 |
|  | Purple | a95 | 839, 840 |
| Corrected exclusions | Yellow | a6 | 90, 92, 94 |
|  | Yellow | a11 | 129, 131, 143 |
|  | Yellow | a22 | 229 |

Four missing studies were identified [1-4]. Clements et al. included their own studies with three-spine stickleback, which live in coastal marine, brackish and freshwater habitats, as well as studies on catadromous eels (study a88) and barramundi (a44, a66, a82) that live in freshwater as adults, therefore the absence of Williams et al. [2] on ocean-phase coho salmon could not be attributed to including only species that are strictly marine. The text and figures in Clements et al. (2022) depict whole years, therefore one study published in March 2019 [3] and one study published in September 2019 [4] were added for completeness.

To perform the reanalysis of the data, the relevant sections of the S1 code file (https://journals.plos.org/plosbiology/article?id=10.1371/journal.pbio.3001511#sec021) was copied into a new R markdown file. The relevant original data files were also downloaded (S2, S5, S7 and S10). The S5, S6 and S7 files all have errors in the columns of publication.online and publication.print, such that the model will not run due to a lack of publications within the year 2009 for publication.online. Unfortunately, it was not a matter of the two columns headings being switched and thus the S2 raw data excel file has been used here to recreate appropriate csv files for analysis (recreation of S5). The S1 file contained code for graphing components of Fig 1B but this did not produce a graph that was visually similar, thus changes and additions to aesthetic related code was made. In addition, the S1 file did not contain the code for Fig 1A, so the code for Fig 1B was modified to allow plotting of visually identical figures, but it may contain slight differences. The same R file was used to run all the reanalyses. Notes in the R markdown file identify where additions or changes were made, all available at Research Data JCU (https://doi.org/10.25903/jw8m-9007).

The data contains experiments with OA treatment levels ranging from <600 to >4000 µatm CO_2_. Because behavioural effects may be weaker and more variable below 800 µatm CO_2_, a separate analysis was done with OA treatments <800 uatm excluded. This excluded 142 of the 839 total cases in the data set, involving studies from 2010-2018. Excluding these cases increased the variance and caused an upward trend in the modelled variance-weighted average effect size for 2010, but otherwise the results were very similar to those with these treatments included (S1 Fig).

References

1. Lechini D, Dixson DL, Lecellier G, Roux N, Frederich B, Besson M, Tanaka Y, Banaigs B, Nakamura Y. Habitat selection by marine larvae in changing chemical environments. Mar Poll Bull. 2017; 114:210-7. doi:10.1016/j.marpolbul.2016.08.083.
2. Williams CR, Dittman AH, McElhany P, Busch DS, Maher MT, Bammler TK, MacDonald JW, Gallagher EP. Elevated CO_2_ impairs olfactory-mediated neural and behavioral responses and gene expression in ocean-phase coho salmon (*Oncorhynchus kisutch*). Global Change Biol. 2019; 25:963-77. doi:10.1111/gcb.14532.
3. Pegado MR, Santos C, Couto A, Pinto E, Lopes AR, Diniz M, Rosa R. Reduced impact of ocean acidification on growth and swimming performance of newly hatched tropical sharks (*Chiloscyllium plagiosum*). Mar Freshwater Behav Physiol. 2018; 51:347-57 (published 28 Mar 2019).
4. Paula JR, Repolho T, Pegado MR, Thörnqvist P-O, Bispo R, Winberg S, Munday PL, Rosa R. Neurobiological and behavioural responses of cleaning mutualisms to ocean warming and acidification. Sci Reports. 2019; 9:12728. doi:10.1038/s41598-019-49086-0 (published 4 Sept 2019).
