# Supplementary material for: Reanalysis shows there is not an extreme decline effect in fish ocean acidification studies
Source: PLoS Biol. 2022 Nov 22;20(11):e3001809. doi: 10.1371/journal.pbio.3001809 (PMC9681065; doi:10.1371/journal.pbio.3001809)
Supplement: S1 Fig — (a) Calculated effect sizes (lnRR) fitted with a Loess curve and 95% confidence bounds and (b) modelled variance–weighted average effect sizes by year. The data underlying this figure may be found in https://doi.org/10.25903/jw8m–9007. Table A. Data errors identified in a non–exhaustive preliminary check of Clements and colleagues’ S2 data file, along with incorrect inclusion of sham treatments, missing data, and exclusions that were corrected to enable analysis. Highlight refers to the colour used to show the relevant lines of data in the screened, corrected, and complete data file used in the reanalysis. Data files available at https://doi.org/10.25903/jw8m–9007. (DOCX) [file pbio.3001809.s002.docx]

Supporting Information: S1 Fig


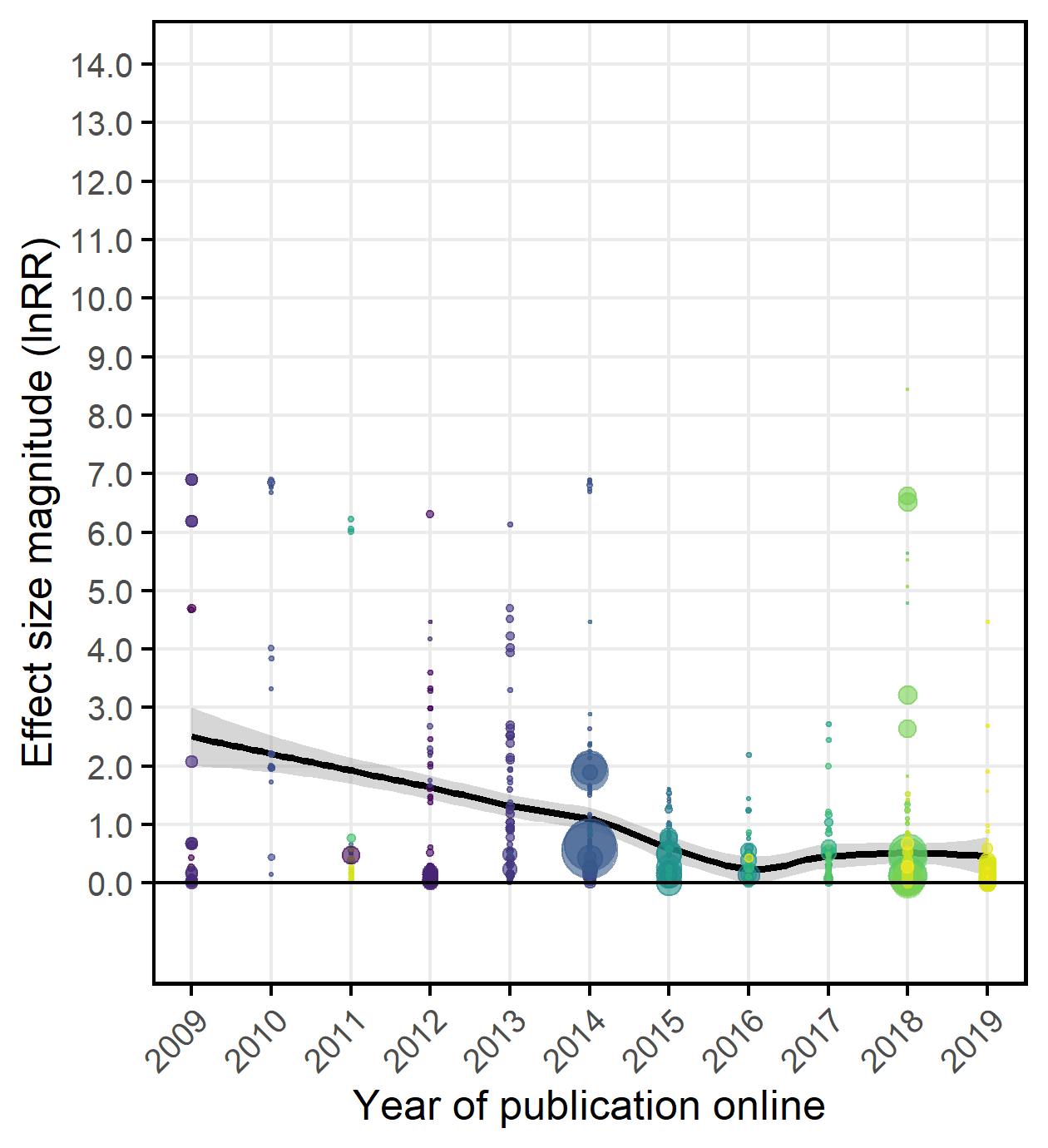

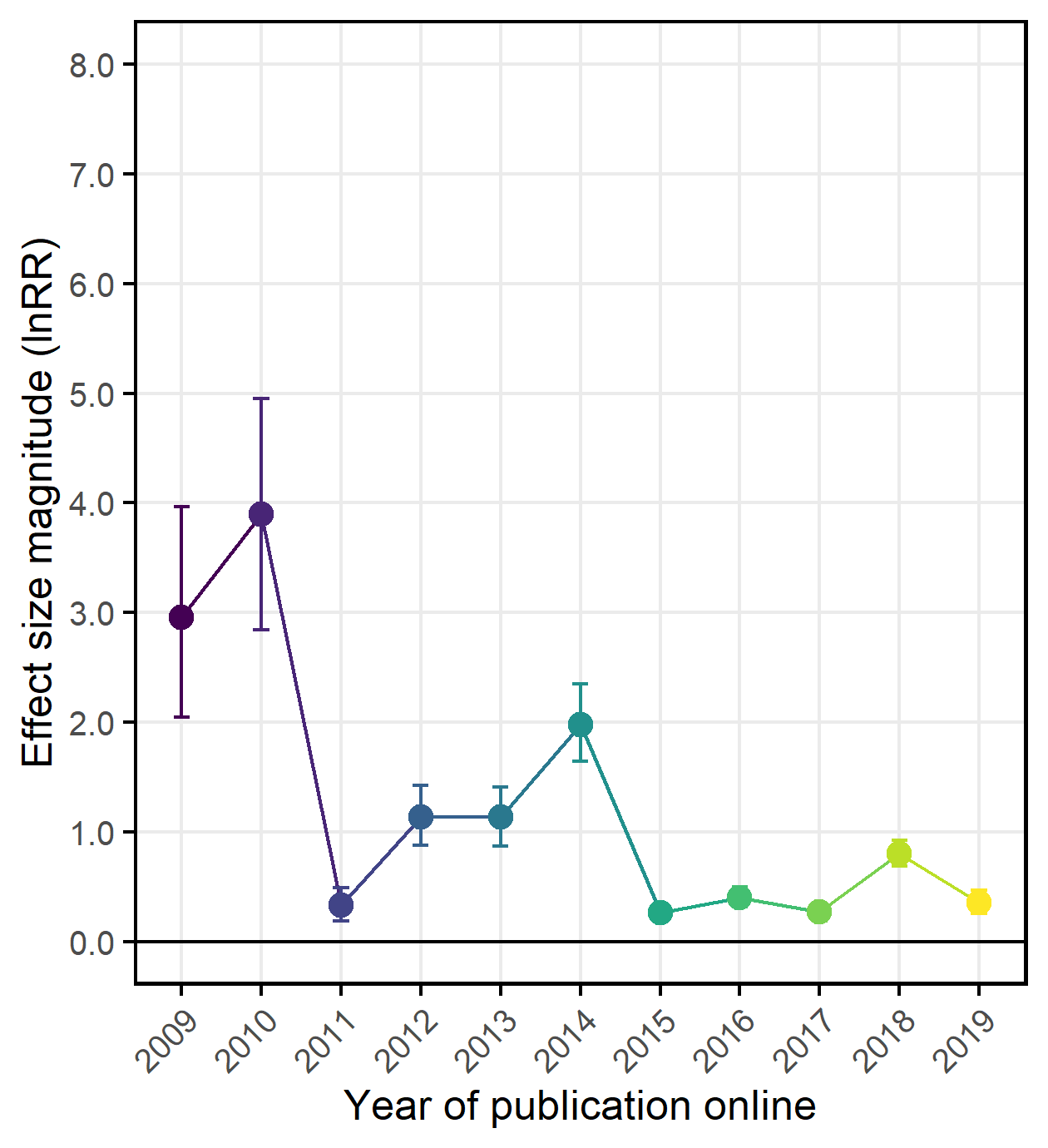


**S1 Fig: Effect sizes in studies on the impacts of ocean acidification on fish behaviour using only OA treatment levels ≥ 800 µatm CO_2_.**

(a) Calculated effect sizes (lnRR) fitted with a Loess curve and 95% confidence bounds and (b) modelled variance-weighted average effect sizes by year. The data underlying this figure may be found in https://doi.org/10.25903/jw8m-9007.
