# Supplementary material for: Reanalysis shows there is not an extreme decline effect in fish ocean acidification studies
Source: PLoS Biol. 2022 Nov 22;20(11):e3001809. doi: 10.1371/journal.pbio.3001809 (PMC9681065; doi:10.1371/journal.pbio.3001809)
Supplement: S1 Data — Calculated lnRR for control values ranging from 0.0001%–5% for treatment values of 50% and 100%. (DOCX) [file pbio.3001809.s003.docx]

Supporting Information: S1 Data

S1 Data: Data associated with Fig 1e. Calculated lnRR for control values ranging from 0.0001 – 5% for treatment values of 50 and 100%.

| Treatment value (%) | Control value (%) | | | | | | | | |
| --- | --- | --- | --- | --- | --- | --- | --- | --- | --- |
|  | 5 | 4 | 3 | 2 | 1 | 0.1 | 0.01 | 0.001 | 0.0001 |
| 50 | 2.3 | 2.5 | 2.8 | 3.2 | 3.9 | 6.2 | 8.5 | 10.8 | 13.1 |
| 100 | 3.0 | 3.2 | 3.5 | 3.9 | 4.6 | 6.9 | 9.2 | 11.5 | 13.8 |
